# Treatment for T1 colorectal cancers substratified by site and size: “horses for courses”

**DOI:** 10.3389/fmed.2023.1230844

**Published:** 2023-10-12

**Authors:** Kexin Shi, Zhen Yang, Kaiming Leng

**Affiliations:** ^1^The Affiliated Hospital of Qingdao University, Qingdao University, Qingdao, China; ^2^Department of Hepatopancreatobiliary Surgery, Qingdao Municipal Hospital, Qingdao University, Qingdao, China; ^3^Qingdao Hospital, University of Health and Rehabilitation Sciences (Qingdao Municipal Hospital), Qingdao, China

**Keywords:** T1 colorectal cancers, characteristics, lymph node metastasis, survival, endoscopic therapy, surgical resection

## Abstract

**Background:**

Owing to advances in diagnostic technology, the diagnosis of T1 colorectal cancers (CRCs) continues to increase. However, the optimal management of T1 CRCs in the Western Hemisphere remains unclear due to limited population-based data directly comparing the efficacy of endoscopic therapy (ET) and surgical resection (SR). The purpose of this study was to report outcome data from a large Western cohort of patients who underwent ET or SR for early CRCs.

**Methods:**

The SEER-18 database was used to identify patients with T1 CRCs diagnosed from 2004 to 2018 treated with ET or SR. Multivariable logistic regression models were employed to identify variables related to lymph node metastasis (LNM). Rates of ET and 1-year relative survival were calculated for each year. Effect of ET or SR on overall survival and cancer-specific survival was compared using Kaplan–Meier method stratified by tumor size and site.

**Results:**

A total of 28,430 T1 CRCs patients were identified from 2004 to 2018 in US, with 22.7% undergoing ET and 77.3% undergoing SR. The incidence of T1 CRCs was 6.15 per 100,000 person-years, with male patients having a higher incidence. Left-sided colon was the most frequent location of tumors. The utilization of ET increased significantly from 2004 to 2018, with no significant change in 1-year relative survival rate. Predictors of LNM were age at diagnosis, sex, race, tumor size, histology, grade, and location. The 5-year relative survival rates were 91.4 and 95.4% for ET and SR, respectively. Subgroup analysis showed that OS and CSS were similar between ET and SR in T1N0M0 left-sided colon cancers with tumors 2 cm or less and in rectal cancers with tumors 1 cm or less.

**Conclusion:**

Our study showed that ET was feasible and safe for patients with left-sided T1N0M0 colon cancers and tumors of 2 cm or less, as well as T1N0M0 rectal cancers and tumors of 1 cm or less. Therefore, the over- and under-use of ET should be avoided by carefully selecting patients based on tumor size and site.

## 1. Introduction

Colorectal cancers (CRCs) rank as the fourth most frequently diagnosed cancer and second leading cause of cancer-related death overall. An estimated 153,020 people in the United States will be diagnosed with CRCs and it will result in the death of 52,550 individuals as of 2023 (1). Although the incidence of CRCs has remained stable or decreased in highly developed countries over the last several decades, recent advances in screening and diagnostic technologies have led to an increase in the detection of early-stage CRCs, including tumors classified as T1 (2). The current treatment options for T1 CRCs include surgical resection (SR) and endoscopic therapy (ET). SR has traditionally been the mainstay of curative intent treatment, but ET is being increasingly adopted for patients with superficial CRCs due to its advantages in reducing treatment-related adverse events, compared to colorectal surgery in clinical practice, especially in the elderly pupulation (3, 4). Several studies have demonstrated that endoscopic removal of tumors is both feasible and suitable for many T1 cancers (5–8). However, ET is technically and clinically challenging due to the varied risks of lymph node metastasis (LNM) among T1 CRCs. Approximately 10% of T1 diseases are found to have LNM at the time of diagnosis, and these patients are candidates for radical surgery. For the remaining patients, endoscopic removal is considered sufficient owing to the low risk of LNM. Moreover, the characteristics of colorectal lesions, such as tumor size, location, and histology, may affect the optimal removal method. Therefore, comparing the long-term survival outcomes of ET and SR is crucial for determining the optimal treatment for T1 CRCs. However, there is a lack of population-based studies that have examined the outcomes of ET versus SR among patients with T1 CRCs in the United States.

Early-stage CRCs present several unresolved clinical questions, including the optimal treatment for T1N0M0 CRCs, which is currently unclear due to the limited population-based data comparing the efficacy of ET and SR in Western hemisphere. Therefore, our study aims to evaluate the relative prevalence, demographics, tumor characteristics, treatment, and survival of patients with T1 CRCs in the United States. In addition, we will analyze the effectiveness of ET versus SR in treating T1 CRCs stratified by tumor size and location.

## Methods

### Data source and patient population

The Surveillance, Epidemiology, and End Results (SEER)-18 registries were used to identify patients with T1 CRCs diagnosed between 2004 and 2018, based on the International Classification of Diseases for Oncology, Third Edition (ICD-O-3). The institutional review board of Qingdao municipal hospital deemed this cohort study exempt from review and informed consent requirement as the data was deidentified and publicly available. And the work has been reported in line with the STROCSS criteria (9).

Patients were then divided into two groups according to their surgical approach used: ET group and SR group. Other studied variables included patient demographic and clinical characteristics. Patient race was coded as white, black, and other. Age at diagnosis was categorized into two groups of <50 years and ≥ 50 years. This age threshold allows us to better capture the disparities in disease characteristics and outcomes between early-onset and late-onset CRCs in our analysis. Tumor characteristics included tumor grade (well-differentiated and poorly-differentiated), size (≤1 cm, ≤2 cm, ≤3 cm, and > 3 cm), histology (adenocarcinoma, mucous, and other), lymph node status (positive and negative), and tumor location (right-sided colon, left-sided colon, and rectum). The primary endpoints were OS and CSS. Patients were excluded if showed a distant metastasis at baseline. And those lacking data on tumor size, surgery, or survival were also not include in our study.

### Statistical analysis

Using the SEER*Stat statistical software, we calculated age-adjusted incidence rates for patients with T1 CRCs overall and specific to sex (male and female) and age groups (0–24, 25–29, 30–34, 35–39, 40–44, 45–49, 50–54, 55–59, 60–64, 65–69, 70–74, 75–79, 80–84, and ≥ 85) from 2004 to 2018. All incidence rates were standardized to the 2000 US standard population and reported per 100,000 person-years. Categorical variables were reported as frequency and percentage, and the distribution of variables between groups were analyzed using *χ*^2^ or Fisher exact tests. Univariate and multivariate logistic regression models were utilized to identify factors associated with lymph node metastasis in T1 CRCs patients. The rates of ET utilization in patients were calculated for each year during the study period of 2004 to 2018. Differences in survival outcomes between ET and SR groups were examined using Kaplan–Meier method with log-rank test. To investigate the effect of surgical approaches on patient survival based on tumor characteristics, we conducted a subgroup analysis stratified by tumor size and site (right-sided colon, left-sided colon, and rectum). Two-sided *p* values and 95% CIs were reported, and *p* < 0.05 was considered as having statistical significance. All statistical analyses were performed by SPSS, version 22.0 and R, version 4.1.1.

## 2. Results

### 2.1. Patient characteristics and incidence

**Figure 1:**
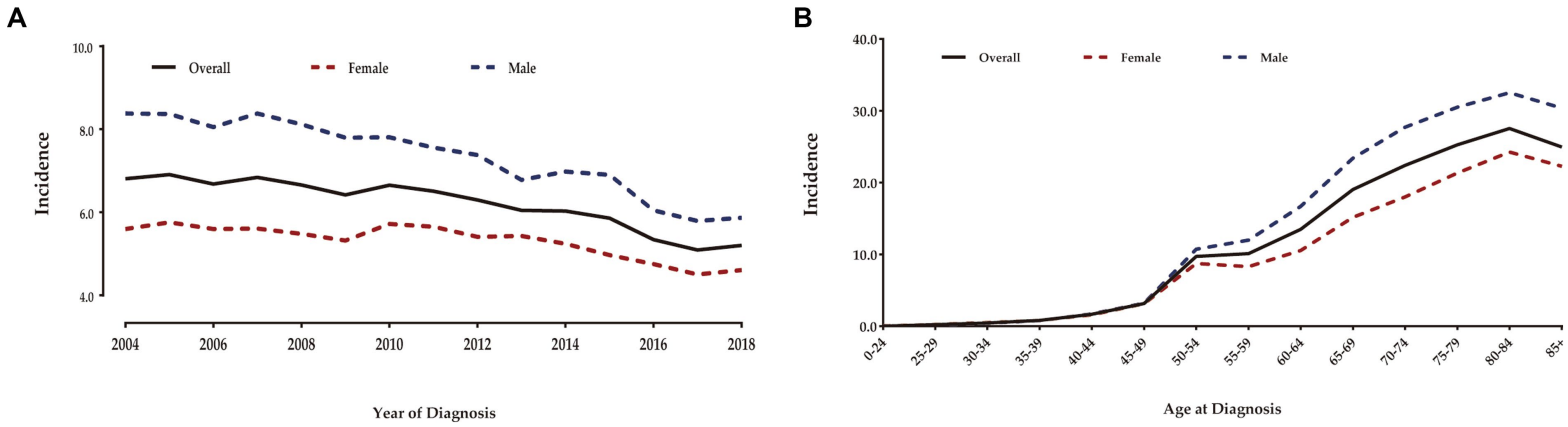
Annual age-adjusted incidence of patients with T1 CRCs in US (Per 100,000 person-years) **(A)**. Age-wise incidence of patients with T1 CRCs in US **(B)**.

After rigorous screening of patients based on inclusion and exclusion criteria, we identified 28,430 eligible patients with T1 CRCs from 2004 to 2018 in the US. Among them, 51.9% were male and the mean age was 64.1 years (SD: 12.6). The majority of patients were White individuals (76.3%) and non-Hispanic (89.7%). LNM was present in only 9.6% of cases. Among these patients, 22.5% underwent ET and 77.5% underwent SR for the tumor. Table 1 shows the detailed demographic and clinical characteristics of all enrolled patients. The overall age-adjusted incidence of T1 CRCs from 2004 to 2018 was 6.15 per 100,000 person-years (Figure 1). Male patients had a higher incidence of T1 CRCs than female patients (7.22 vs. 5.26 per 100,000 person-years). The incidence increased with age, with the highest incidence occurring between 80–84 years old for both male and female patients.

**Table 1:** Characteristics and survival of patients with T1 CRC between 2004 and 2018.

Variables	No. of patients (%)
Age (years)	
<50	2841 (10.0)
≥50	25589 (90.0)
Mean, y (SD)	64.1 (12.6)
Sex	
Male	14763 (51.9)
Female	13667 (48.1)
Race	
White	21699 (76.3)
Black	3426 (12.1)
Other	3305 (11.6)
Ethnicity	
Non-Hispanic	25504 (89.7)
Hispanic	2926 (10.3)
Marital status	
Married	16539 (58.2)
Other	11891 (41.8)
Year of diagnosis	
2004–2008	7431 (26.1)
2009–2013	9043 (31.8)
2014–2018	11956 (42.1)
Tumor size	
≤ 1 cm	11848 (41.7)
≤ 2 cm	8197 (28.8)
≤ 3 cm	4356 (15.3)
> 3 cm	4029 (13.2)
Histology	
Adenocarcinoma	27616 (97.1)
Mucous	653 (2.3)
Other	161 (0.6)
Grade	
Well differentiated	26382 (92.8)
Poorly differentiated	2048 (7.2)
Location	
Right-sided colon	9771 (34.4)
Left-sided colon	10732 (37.7)
Rectum	7927 (27.9)
Lymph node metastasis	
No	25696 (90.4)
Yes	2734 (9.6)
Surgery	
ET	6403 (22.5)
SR	22027 (77.5)
Chemotherapy	
No	25501 (89.7)
Yes	2929 (10.3)
Radiation	
No	26950 (94.8)
Yes	1480 (5.2)
Overall survival	
Mean (95% CI)	137.8 (136.9–138.7)

CRC, colorectal cancer; CI, confidence interval.

### Tumor characteristics and treatment

**Figure 2:**
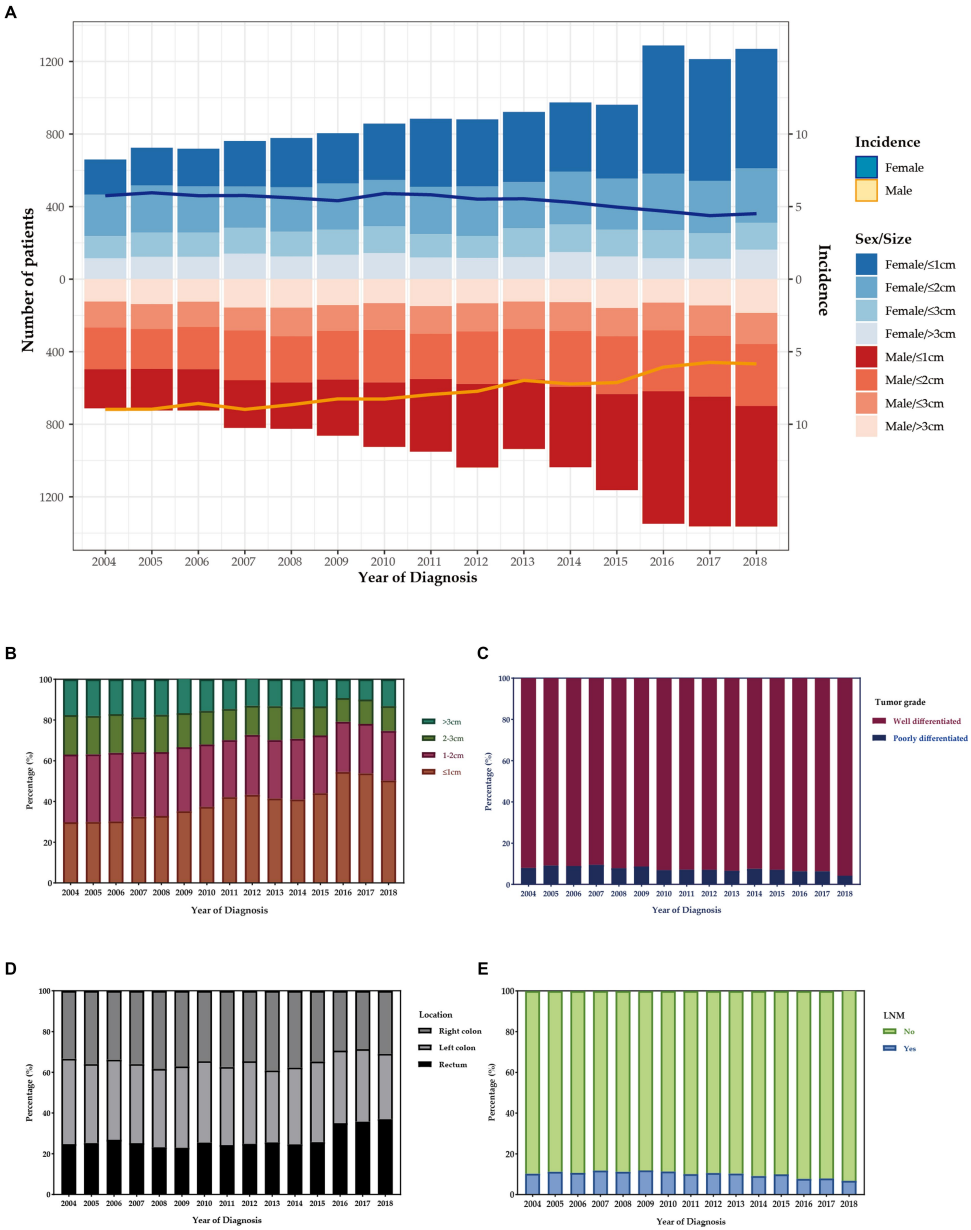
**(A)** The age distribution of cases with T1 CRCs from 2004 to 2018 in US. The bar was the number of cases in females and males, and the line with 95% CI represents age-adjusted incidence. Trends of distribution in tumor size **(B)**, differentiation **(C)**, location **(D)**, and LNM **(E)** over the study period.

**Figure 3:**
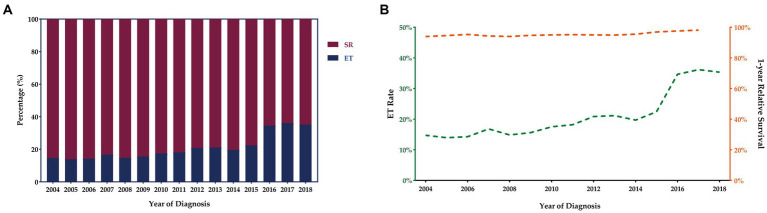
Trends in treatment modalities for T1 CRCs in US from 2004 to 2018 **(A)**. ET rates and 1-year relative survival rate for patients with T1 CRCs from 2004 to 2018 **(B)**.

The number of T1 CRCs patients increased with year synchronously in males and females, with the most pronounced increase appearing in tumors that were 1 cm or smaller (Figure 2A). Most tumors diagnosed were well-differentiated (92.8%), while the remaining 7.2% were poorly differentiated (Table 1). The left-sided colon was the most common location of tumors (37.7%), followed by the right-sided colon (34.4%) and rectum (27.9%). Figure 2 illustrates the trends in tumor size, differentiation, location, and LNM over the study period from 2004 to 2018. The proportion of patients with LNM remained relatively constant throughout the study period. However, there was a significant increase in the percentage of patients receiving ET during the follow-up period, from 14.7% in 2004 to 35.3% in 2018. Despite this, there was no significant change in the 1-year relative survival rate of T1 CRCs patients between 2004 and 2018 (Figure 3). These findings suggest that ET is increasingly being used as a treatment option for T1 CRCs patients.

### Predictors of LNM

There were 2734 out of 28487 patients (9.6%) with T1 CRCs who were found to have LNM. The odds of LNM in patients with T1 CRCs were analyzed by a logistic regression model. Factors associated with LNM in multivariate analysis were age at diagnosis [≥50 (OR: 0.68, 95%CI: 0.60–0.77, *p* < 0.001)], sex [male (OR: 0.86, 95%CI: 0.80–0.94, *p* < 0.001)], race [Black (OR: 1.19, 95%CI: 1.05–1.34, *p* = 0.005)], tumor size [size≤2 cm (OR: 1.64, 95%CI: 1.48–1.82, *p* < 0.001), size≤3 cm (OR: 1.73, 95%CI: 1.53–1.95, *p* < 0.001), size>3 cm (OR: 2.25, 95%CI: 2.01–2.53, *p* < 0.001)], histology [mucous (OR: 1.86, 95%CI: 1.52–2.29, *p* < 0.001)], tumor grade (OR: 2.46, 95%CI: 2.18–2.77, *p* < 0.001), and location [left-sided colon (OR: 1.39, 95%CI: 1.27–1.53, *p* < 0.001)] (Table 2).

**Table 2:** Factors associated with LNM among patients with T1 CRCs.

Variables	Univariate analysis		Multivariate analysis	
	OR (95% CI)	*p* value	OR (95% CI)	*p* value
Age				
< 50	Ref		Ref	
≥ 50	0.67 (0.59, 0.75)	**<0.001**	0.68 (0.60, 0.77)	**<0.001**
Sex				
Female	Ref		Ref	
Male	0.86 (0.80, 0.93)	**<0.001**	0.86 (0.80, 0.94)	**<0.001**
Race				
White	Ref		Ref	
Black	1.15 (1.03, 1.30)	**0.017**	1.19 (1.05, 1.34)	**0.005**
Other	1.04 (0.92, 1.17)	0.560	1.08 (0.95, 1.22)	0.256
Ethnicity				
Non-Hispanic	Ref			
Hispanic	1.00 (0.88, 1.14)	0.967		
Tumor size				
≤ 1 cm	Ref		Ref	
≤ 2 cm	1.71 (1.54, 1.89)	**<0.001**	1.64 (1.48, 1.82)	**<0.001**
≤ 3 cm	1.77 (1.58, 2.00)	**<0.001**	1.73(1.53, 1.95)	**<0.001**
> 3 cm	2.24 (2.00, 2.52)	**<0.001**	2.25 (2.01, 2.53)	**<0.001**
Histology				
Adenocarcinoma	Ref		Ref	
Mucous	2.17 (1.77, 2.66)	**<0.001**	1.86 (1.52, 2.29)	**<0.001**
Other	1.14 (0.69, 1.88)	0.617	0.76 (0.45, 1.27)	0.297
Tumor grade				
Well differentiated	Ref		Ref	
Poorly differentiated	2.58 (2.30, 2.90)	**<0.001**	2.46 (2.18, 2.77)	**<0.001**
Location				
Right-sided colon	Ref		Ref	
Left-sided colon	1.31 (1.20, 1.43)	**<0.001**	1.39 (1.27, 1.53)	**<0.001**
Rectum	0.88 (0.79, 0.97)	**0.015**	0.91 (0.82, 1.02)	0.104

LNM, lymph node metastasis, CRCs, colorectal cancers, OR, odds ratio, CI, confidence interval, Ref: reference. Bold indicates significance.

### 2.2. Survival

**Figure 4:**
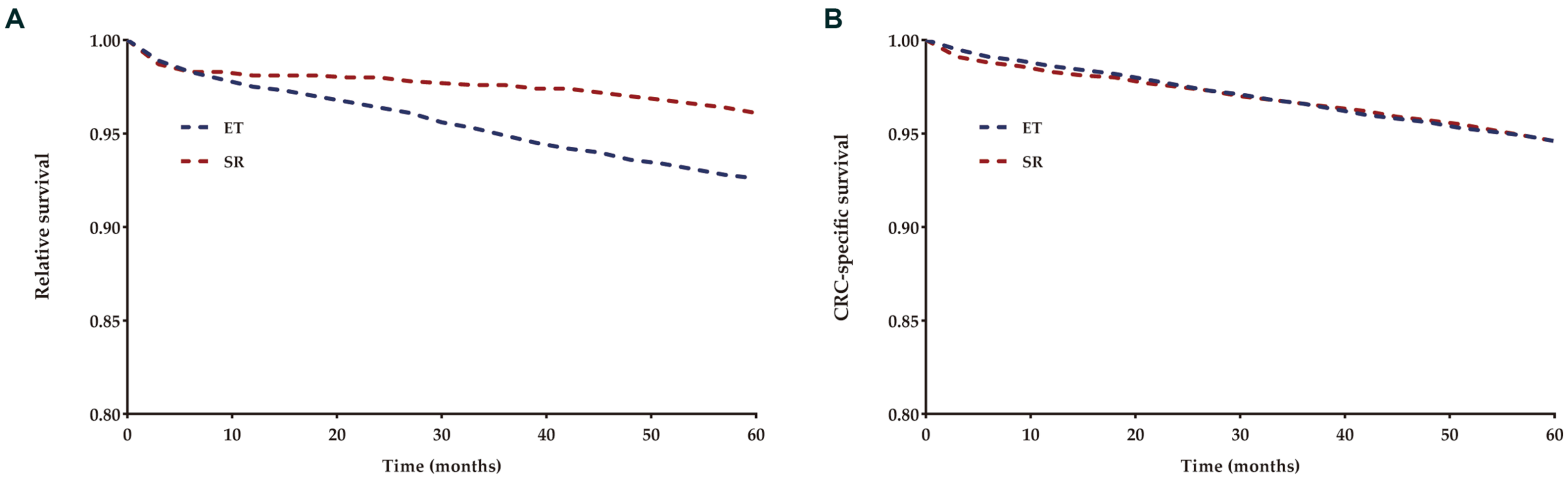
Relative **(A)** and cancer-specific **(B)** survival for each treatment group compared to US population.

The 5-year relative survival (RS) rates for T1 CRCs were 91.4 and 95.4% for patients who underwent ET and SR, respectively, while the 5-year cause-specific survival (CSS) rates were 94.6% for both groups (Figure 4).

### 2.3. Subgroup analysis according to tumor size

**Figure 5:**
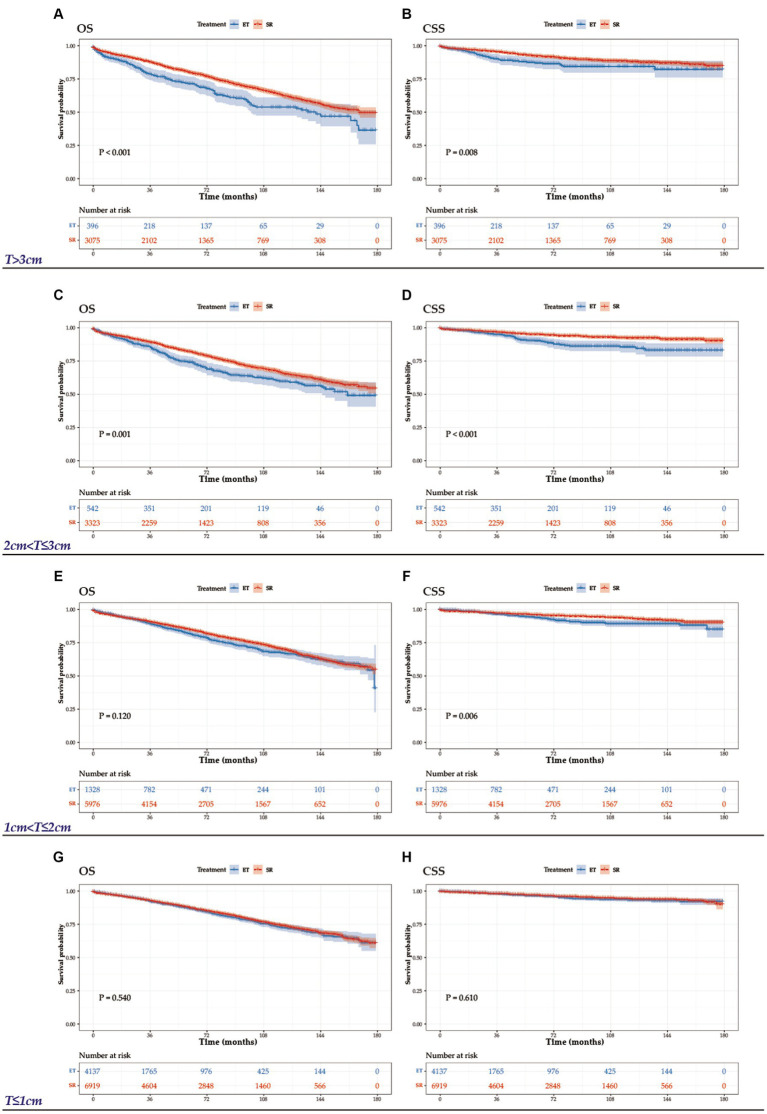
OS and CSS graphs by treatment for tumors >3 cm **(A, B)**, 2 cm < T ≤ 3 cm **(C, D)**, 1 < T ≤ 2 cm **(E, F)**, and T ≤ 1 cm **(G, H)**.

The study included 25696 patients with T1N0M0 CRCs, classified into four groups based on tumor size: T ≤ 1 cm (*n* = 11056), 1 cm < T ≤ 2 cm (*n* = 7304), 2 cm < T ≤ 3 cm (*n* = 3865), and > 3 cm (*n* = 3471). The baseline characteristics of all patients, stratified by treatment group, are presented in Table 3. Significant differences were observed in all clinical features between these two cohorts. As expected, patients with small (≤1 cm) and rectal cancer were more likely to receive ET. During the follow-up period, there were 1005 deaths among the 6403 patients in the ET group and 3996 deaths among the 19293 patients in the SR group. To determine the effect of ET on OS and CSS, we analyzed the survival outcomes using the Kaplan–Meier method (Figure 5). Our analysis revealed that OS was comparable between patients who received ET and SR for tumors 2 cm or less (1 cm < T ≤ 2 cm: HR, 0.90, 95%CI, 0.79–1.03, *p* = 0.120; ≤1 cm: HR, 0.97, 95%CI, 0.87–1.08, *p* = 0.540), but SR was associated with significantly better OS in tumors larger than 2 cm (>3 cm: HR, 0.69, 95%CI, 0.57–0.83, *p* < 0.001; 2 cm < T ≤ 3 cm: HR, 0.75, 95%CI, 0.63–0.89, *p* = 0.001). Meanwhile, for CSS, there was no significant difference between ET and SR in tumors 1 cm or less (HR, 0.94, 95%CI, 0.75–1.18, *p* = 0.610), but SR was associated with better CSS in patients with tumors larger than 1 cm (>3 cm: HR, 0.65, 95%CI, 0.47–0.90, *p* = 0.008; 2 cm < T ≤ 3 cm: HR, 0.52, 95%CI, 0.38–0.71, *p* < 0.001; 1 cm < T ≤ 2 cm: HR, 0.70, 95%CI, 0.55–0.91, *p* = 0.006).

**Table 3:** Comparison of baseline characteristics of T1N0M0 CRCs by treatment group.

Variables	ET (*n* = 6403)	SR (*n* = 19293)	*p* value
Mean age, y (SD)	61.7 (13.1)	65.2 (12.4)	**<0.001**
Age, y			**<0.001**
< 60	3030 (47.3%)	6377 (33.1%)	
≥ 60	3373 (52.7%)	12916 (66.9%)	
Sex			**<0.001**
Male	3480 (54.3%)	9955 (51.6%)	
Female	2923 (45.7%)	9338 (48.4%)	
Race			**<0.001**
White	4497 (70.2%)	15157 (78.6%)	
Black	881 (13.8%)	2178 (11.3%)	
Other	1025 (16.0%)	1958 (10.1%)	
Ethnicity			**<0.001**
Non-Hispanic	5670 (88.6%)	17382 (90.1%)	
Hispanic	733 (11.4%)	1911 (9.9%)	
Marital status			**<0.001**
Married	3473 (54.2%)	11375 (59.0%)	
Other	2930 (45.8%)	7918 (41.0%)	
Year of diagnosis			**<0.001**
2004–2008	1099 (17.2%)	5522 (28.6%)	
2009–2013	1681 (26.3%)	6398 (33.2%)	
2014–2018	3623 (56.5%)	7373 (38.2%)	
Tumor size			**<0.001**
≤ 1 cm	4137 (64.6%)	6919 (35.9%)	
≤ 2 cm	1328 (20.7%)	5976 (31.0%)	
≤ 3 cm	542 (8.5%)	3323 (17.2%)	
> 3 cm	396 (6.2%)	3075 (15.9%)	
Histology			**<0.001**
Adenocarcinoma	6253 (97.7%)	18766 (97.3%)	
Mucous	66 (1.0%)	467 (2.4%)	
Other	84 (1.3%)	60 (0.3%)	
Grade			**<0.001**
Well differentiated	6111 (95.4%)	17946 (93.0%)	
Poorly differentiated	292 (4.6%)	1347 (8.0%)	
Location			**<0.001**
Right-sided colon	371 (5.8%)	8523 (44.2%)	
Left-sided colon	1990 (31.1%)	7515 (39.0%)	
Rectum	4042 (63.1%)	3255 (16.8%)	
Chemotherapy			0.339
No	6141 (95.9%)	18555 (96.2%)	
Yes	262 (4.1%)	738 (3.8%)	
Radiation			**<0.001**
No	6063 (94.7%)	18610 (96.5%)	
Yes	340 (5.3%)	683 (3.5%)	

ET, endoscopic therapy; SR, surgical resection. Bold indicates significance.

### Subgroup analysis according to tumor site

Table 4 presents the 3-year OS and CSS rates of patients with T1N0M0 CRCs categorized by tumor location. The results of the site-specific analysis indicate that for patients with left-sided colon cancer and tumors 2 cm or less, as well as for patients with rectal cancer and tumors 1 cm or less, the survival outcomes including OS and CSS were similar between ET and SR treatment options.

**Table 4:** Subgroup survival analysis between ET and SR.

Tumor location	Size	3-Year OS		*p* value	3-Year CSS		*p* value
		ET	SR		ET	SR	
Right-sided colon	T > 3 cm	49.4%	86.5%	<0.001	65.7%	95.1%	<0.001
	2 cm < T ≤ 3 cm	78.1%	87.7%	0.001	89.1%	97.0%	0.005
	1 cm < T ≤ 2 cm	73.0%	88.8%	0.007	92.1%	96.7%	0.027
	T ≤ 1 cm	79.7%	91.5%	<0.001	97.2%	97.9%	0.423
Left-sided colon	T > 3 cm	83.4%	88.5%	0.035	94.6%	96.3%	0.598
	2 cm < T ≤ 3 cm	84.4%	89.4%	0.018	96.0%	96.8%	0.312
	1 cm < T ≤ 2 cm	91.6%	91.5%	0.311	98.0%	97.4%	0.345
	T ≤ 1 cm	92.4%	93.0%	0.271	98.0%	98.3%	0.451
Rectum	T > 3 cm	80.2%	89.3%	<0.001	91.5%	94.6%	0.633
	2 cm < T ≤ 3 cm	86.3%	92.5%	<0.001	95.3%	96.7%	0.045
	1 cm < T ≤ 2 cm	89.6%	93.8%	<0.001	96.0%	97.8%	0.009
	T ≤ 1 cm	93.4%	93.5%	0.933	97.8%	96.6%	0.063

OS, overall survival; CSS, cancer-specific survival; ET, endoscopic therapy; SR, surgical resection.

## Discussion

Our study provides a comprehensive analysis on the topic of T1 CRCs, including examining the epidemiology, clinicopathologic characteristics, treatment, and survival from 2004 to 2018 in the United States. To the best of our knowledge, this study is the first to explore two key aspects of T1 CRCs using the SEER-18 database: (1) the epidemiology of T1 CRCs and (2) the subclassifications of treatment modalities for T1N0M0 CRCs based on tumor size and site. We found that the incidence of T1 CRCs was 6.15 per 100,000 person-years, with higher rates observed in males than females (7.22 vs. 5.26 per 100,000 person-years). Incidence increased with age and peaked between ages 80–84 for both genders. Additionally, 9.6% of the patients were found to have LNM. Factors associated with LNM in multivariate analysis were age at diagnosis, sex, race, tumor size, histology, tumor grade, and location. Our findings also suggest an increasing trend in the use of ET as a treatment option for T1 CRCs patients. However, subgroup analysis according to tumor size and site demonstrates that ET was associated with similar survival outcomes to SR only in T1N0M0 patients with left-sided colon cancer and tumors 2 cm or less or in patients with rectal cancer and tumors 1 cm or less.

With the advancement of colonoscopic techniques, endoscopic removal of neoplasms has become one of the preferred treatments for T1 disease. However, the presence of LNM is a crucial factor in determining the feasibility and suitability of ET, as it is only safe for patients in the absence of nodal metastasis. Our study found that LNM occurred in 9.6% of T1 CRCs patients, consistent with previous studies, indicating that nearly 90% of this population can benefit from ET (10–12). Identifying those at low risk of LNM could help balance the potential risks and benefits of ET for treating early-stage CRCs. Multivariable analyses in our study revealed that age, sex, race, tumor size, histology, tumor differentiation, and location were significantly associated with LNM in patients with submucosal invasive CRCs. This information can be used to make evidence-based decisions regarding active surveillance or additional radical resection following endoscopic resection. While previous studies have identified several pathological high-risk features, such as deep submucosal invasion, lymphovascular invasion, positive resection margin, and tumor budding, as predictors of presence of LNM in early CRCs, our study could not investigate these factors due to limitations of the SEER database (13–15). Accurately evaluating the risk of LNM is critical in determining whether further radical resection is necessary for patients who have undergone ET. Kudo et al. developed an algorithm using artificial intelligence that includes patients’ age, tumor size, grade, location, lymphatic invasion, and vascular invasion to identify those with T1 CRCs who are at higher risk for LNM (16). This model could help in providing appropriate care without excess or deficient treatment for patients. Future studies should investigate the associations between patient characteristics and LNM to provide more comprehensive guidance for clinical decision-making.

Our study also found a significant increase in the use of ET for T1 CRCs in US from 2004 to 2018, with utilization rates rising from 14.7 to 35.3%. This trend may reflect the growing acceptance of ET as a feasible alternative to SR for treating early-stage invasive CRCs, likely driven by improving endoscopic techniques as well as the higher incidence of T1 disease as a result of population-based screening programs. Similar trends have been observed in the treatment of early-stage esophageal and gastric cancers (17, 18). Considering the increasing detection of CRCs at early stages, we are not surprised to find that the utilization of endoscopic removal for tumors will continue to rise in the future.

Most published studies on the outcomes of ET versus SR for early gastrointestinal cancers have been conducted in Asian countries (19–22). Therefore, it is essential to validate these findings in the Western population. Our study, based on a population-based registry from US, suggests that ET may be a promising treatment option for T1N0M0 left-sided colon cancers of 2 cm or less and T1N0M0 rectal cancers of 1 cm or less. A systematic review and meta-analysis by Yeh et al. involving 17 studies and 19,979 patients with colorectal cancers demonstrated comparable long-term survival outcomes between ET and SR for T1 CRCs (23). Additionally, Jang et al. utilized a Markov model to assess the cost-effectiveness of various treatment strategies in T1 CRCs based on biomarker profiles and concluded that ET was more suitable for patients with less aggressive biomarkers (24). Refinements in the endoscopic technology have significantly improved the management of colorectal lesions. However, one of the main challenges in treating early-stage CRCs is choosing the most suitable method of ET that ensures complete resection, reduces recurrence risk, and mitigates complications. A large meta-analysis, comprising 50 studies and involving 6,442 patients, was carried out to assess the effectiveness and safety of ET, and the results revealed that ET emerged as an exceedingly effective and safe intervention (25). It achieved an initial success rate of 92%, with only 8% of patients ultimately undergoing surgery due to the inadequacy of endoscopic resection for cure. It’s worth noting that the incidence of perforation and delayed bleeding stood at 1.5 and 6.5%, respectively. These results collectively underscore the remarkable effectiveness and safety profile of ET in clinical practice. Endoscopic mucosal resection (EMR) and endoscopic submucosal dissection (ESD) are two main minimally invasive techniques used to remove superficially invasive CRCs, with ESD facilitating a higher en-bloc resection rate regardless of tumor size (26). However, ESD is associated with certain drawbacks. It is a technically demanding and time-consuming procedure, necessitating specialized training and dedicated equipment. Moreover, ESD entails elevated costs and poses higher risks of bleeding and perforation, especially when applied to colonic cases. Consequently, its execution should be entrusted to experienced gastroenterologists and surgeons operating in well-equipped centers with rigorous quality control protocols. Regrettably, ESD adoption remains relatively scarce in Western countries. A recent meta-analysis, encompassing 238 publications published between 1990 and 2016, investigated the efficacy of ESD on a global scale. Of note, 90% of the included studies originated from Eastern countries, while only 10% were conducted in Western countries (27). The findings revealed that ESD outcomes were significantly superior in Eastern countries compared to Western countries, prompting the authors to emphasize the importance of considering local ESD expertise and regional outcomes when treating gastrointestinal lesions with ESD. Similarly, another meta-analysis involving 97 studies, with 71 conducted in Asia, indicated that the standard ESD technique in non-Asian countries is still falling short in achieving satisfactory performance levels (28). In a retrospective analysis of ESD procedures performed in a high-volume US referral center, Zhang et al. observed that Western practitioners commonly faced a longer learning curve in comparison to their Asian counterparts. This disparity may be attributed in part to the limited availability of experienced trainers and training programs, as well as scarcity of easier lesions (29, 30). Prior studies showed that ESD for CRCs in the western countries achieves en-bloc lesion resection removal in 50–84% and curative resection rate in 74–92%, with a perforation and bleeding rate of 1.3–20% and 7.9–12%, respectively, which is not as favorable as in Eastern hemisphere studies (31–33). Given these limitations, there is a pressing need for developing an endoscopic technique superior to conventional EMR in terms of en bloc resection and local recurrence prevention, while minimizing the occurrence of adverse events. In this context, Underwater endoscopic mucosal resection (UEMR) has emerged as a compelling and oncologically safe alternative to EMR for the treatment of colorectal cancers (34–40). The findings reported by Nagl et al. are valuable additions to the literature, as they suggest that UEMR could serve as an intermediate approach, bridging the gap between smaller lesions suitable for conventional EMR and larger lesions where ESD might be the preferred procedure (36). Further research is warranted to compare the long-term outcomes and cost-effectiveness of various ET methods and to identify the optimal management approach for T1 CRCs.

In the context of our primary focus on comparing the efficacy of ET and SR, it’s worth noting that studies conducted by Marcellinaro and colleagues shed light on potential avenues for improving postoperative care and reducing complications in surgical interventions for CRCs. Marcellinaro et al. reported encouraging results with the Microbiota Implementation to Reduce Anastomotic Colorectal Leaks (MIRACLe) protocol in patients undergoing colorectal surgery for cancer (41). The MIRACLe protocol significantly reduced anastomotic leaks (AL), with an incidence as low as 1.7% in the post-matched MIRACLe group compared to 6.5% in the post-matched Control group. Similarly, a pilot study by the same group observed positive trends with the MIRACLe protocol, with a 1.7% AL incidence in the MIRACLe group compared to 6.4% in the control group (42). These findings suggest the potential benefits of microbiota manipulation as a complementary strategy to enhance surgical outcomes in CRCs patients.

It is important to acknowledge the limitations of our study, which include the reliance on data from the SEER database, limited availability of detailed clinical information, and the absence of prospective clinical trials for validation. The SEER database did not provide information regarding medical comorbidities, complications, lymphovascular invasion, margin status, or disease recurrence. Additionally, the lack of data on ulceration prevented us from determining the effect of this factor on long-term survival outcomes in patients after ET or SR. Patients who were ill or older were more likely to undergo ET due to its less invasive nature. As this was a retrospective study, inherent selection biases could not be fully avoided.

Our study revealed an increasing utilization of ET and promising survival outcomes for patients with T1 CRCs in US, which corresponds with the evolution of endoscopic techniques. Further analysis showed that ET was feasible and safe for patients with left-sided T1 colon cancers and tumors of 2 cm or less, as well as T1 rectal cancers and tumors of 1 cm or less. Therefore, the over- and under-use of ET should be avoided by carefully selecting patients based on tumor size and site. Future studies are required to examine the effectiveness of EMR and ESD at a population level in the Western hemisphere.

## Data availability statement

The datasets presented in this study can be found in online repositories. The names of the repository/repositories and accession number(s) can be found in the article/supplementary material.

## Author contributions

KL: conception. KS: data analysis. ZY: writing. All authors contributed to the article and approved the submitted version.
